# Plant Species Richness and the Root Economics Space Drive Soil Fungal Communities

**DOI:** 10.1111/ele.70032

**Published:** 2024-12-31

**Authors:** Justus Hennecke, Leonardo Bassi, Cynthia Albracht, Angelos Amyntas, Joana Bergmann, Nico Eisenhauer, Aaron Fox, Lea Heimbold, Anna Heintz‐Buschart, Thomas W. Kuyper, Markus Lange, Yuri Pinheiro Alves de Souza, Akanksha Rai, Marcel Dominik Solbach, Liesje Mommer, Alexandra Weigelt

**Affiliations:** ^1^ Systematic Botany and Functional Biodiversity, Institute of Biology Leipzig University Leipzig Germany; ^2^ German Centre for Integrative Biodiversity Research (iDiv) Halle‐Jena‐Leipzig Leipzig Germany; ^3^ Biosystems Data Analysis, Swammerdam Institute for Life Sciences University of Amsterdam Amsterdam the Netherlands; ^4^ Department Soil Ecology Helmholtz Centre for Environmental Research—UFZ Halle (Saale) Germany; ^5^ Institute for Biosafety in Plant Biotechnology Julius Kühn Institute Quedlinburg Germany; ^6^ Institute of Biodiversity Friedrich Schiller University Jena Jena Germany; ^7^ J.F. Blumenbach Institute of Zoology and Anthropology University of Göttingen Göttingen Germany; ^8^ Leibniz Centre for Agricultural Landscape Research (ZALF) Müncheberg Germany; ^9^ Institute of Biology Leipzig University Leipzig Germany; ^10^ Chair of Environmental Microbiology TUM School of Life Science, Technical University of Munich Freising Germany; ^11^ Environment, Soils and Land Use, Teagasc, Johnstown Castle Co. Wexford Ireland; ^12^ Institute of Biology Martin‐Luther‐University Halle‐Wittenberg Halle (Saale) Germany; ^13^ Soil Biology Group Wageningen University Wageningen The Netherlands; ^14^ Department of Biogeochemical Processes Max Planck Institute for Biogeochemistry Jena Germany; ^15^ Research Unit Comparative Microbiome Analysis Helmholtz Zentrum München Neuherberg Germany; ^16^ Terrestrial Ecology Group, Institute of Zoology University of Cologne Cologne Germany; ^17^ Forest Ecology and Forest Management Group Wageningen University & Research Wageningen The Netherlands

**Keywords:** arbuscular mycorrhizal fungi, collaboration gradient, pathogenic fungi, plant–fungi interactions, root economics space, root traits, saprotrophic fungi, trait‐based

## Abstract

Trait‐based approaches have been increasingly used to relate plants to soil microbial communities. Using the recently described root economics space as an approach to explain the structure of soil‐borne fungal communities, our study in a grassland diversity experiment reveals distinct root trait strategies at the plant community level. In addition to significant effects of plant species richness, we show that the collaboration and conservation gradient are strong drivers of the composition of the different guilds of soil fungi. Saprotrophic fungi are most diverse in species‐rich plant communities with ‘slow’ root traits, whereas plant pathogenic fungi are most diverse and abundant in communities with ‘fast’ and ‘DIY’ root traits. Fungal biomass is strongly driven by plant species richness. Our results illustrate that the root economics space and plant species richness jointly determine the effects of plants on soil fungal communities and their potential role in plant fitness and ecosystem functioning.

## Graphical abstract

Soil fungi are critical for plants and ecosystems but are also affected by the vegetation itself. Here, we show how an experimental gradient of plant species richness and community fine root traits drive the diversity and abundance of soil fungal guilds. We highlight that fungal biomass is determined by plant biomass and plant species richness, whereas the diversity and relative abundance of individual fungal guilds are linked to the fine root traits of the plant community.

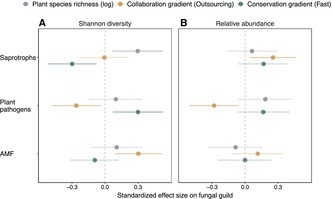

## 1 Introduction

Soil fungi can act as mutualists or antagonists of plants and thus promote or reduce the functioning of plants and ecosystems (Tedersoo et al. 2014; Tedersoo, Bahram, and Zobel 2020; Wagg et al. 2014). Understanding the drivers of the guild composition of fungal communities is important for the understanding of ecological processes that shape plant communities and is crucial for the sustainable management of soils (Jansson, McClure, and Egbert 2023; Lutz et al. 2023; Schmidt, Mitchell, and Scow 2019). Plant communities can exert a strong selective pressure on soil fungal communities (Scherber et al. 2010; Zak et al. 2003). Both plant species richness (Zak et al. 2003) and composition (Bever et al. 2010; Reynolds et al. 2003) and, therefore, the associated plant traits are important components of belowground plant–fungal relationships. In the last decades, trait‐based approaches have emerged to give valuable insights into plants as drivers of the soil microbial community (Bergmann et al. 2020; Wardle et al. 2004) but these largely lack a belowground trait perspective.

Root traits are not just analogues of leaf traits (Bergmann et al. 2017); their variation along two orthogonal axes has been described in the so‐called root economics space (RES) (Bergmann et al. 2020). Roots, similar to leaves, vary in tissue density and relative nitrogen content along the ‘fast–slow’ axis of the conservation gradient. Because root nitrogen concentrations are closely linked to root metabolic activity, relative growth rate and root longevity (Hou et al. 2024; Poorter et al. 1991; Reich et al. 2008; Zhou et al. 2018), roots with high nitrogen content are considered ‘fast’, in the context of both root economics and whole plant economics (Weigelt et al. 2021). However, there is an additional trade‐off between specific root length (SRL) and mean root diameter (Bergmann et al. 2020). This trade‐off has been explained by the interaction between roots and their associated arbuscular mycorrhizal fungi (AMF), with thicker roots generally hosting more AMF (‘outsourcing’ strategy) and thinner roots maximising root surface for independent nutrient uptake (‘do‐it‐yourself’, ‘DIY’ strategy) (Bergmann et al. 2020; Kong et al. 2014). Recent studies have largely confirmed this global trait coordination of species across regions and vegetation types (Han and Zhu 2021; Hennecke et al. 2023; Spitzer et al. 2021). However, it is unclear to what extent the species‐level RES is also represented at the plant community level (Da et al. 2023; Lachaise et al. 2022). Therefore, understanding how root functional strategies scale from the species level to the community level is critical to utilising the RES as a trait‐based framework for soil microbial communities and ecosystem functioning.

Multiple components of the fungal community can be affected by abiotic and biotic factors. Based on a functional classification, soil fungal communities are composed of three main guilds: saprotrophic, pathogenic and mycorrhizal fungi (Põlme et al. 2021). While the overall fungal abundance (or biomass) can change, e.g., through increased resource availability (Waldrop et al. 2006), changes in the functional or taxonomic composition of the community likely result from interspecific processes such as competition or differences in resource utilisation among taxa or guilds (Albornoz et al. 2022). Approaches integrating relative (i.e., compositional) and absolute measures of fungal communities are needed for a holistic view of the effects of plants on fungal communities, especially as both components are relevant to ecosystem processes (Graham et al. 2016). Generally, soil fungal communities are closely linked to the plant community, as they are not only largely dependent on carbon input from the plant but also drive the nutrient cycling and availability to the plant (van der Heijden, Bardgett, and van Straalen 2008). However, frameworks that explicitly link plant traits to the functional composition of soil fungal communities are limited in number and explanatory power (Barberán et al. 2015; Ferlian, Wirth, and Eisenhauer 2017), and studies that empirically tested trait‐based frameworks have largely omitted root traits (de Vries et al. 2012). The additional complexity of trait variation in roots compared to leaves is not yet integrated into many trait‐based approaches but could offer valuable potential to better understand plant communities as drivers of soil fungal communities.

If the RES exists at the community level, it can be used to extend the initial framework of Wardle et al. (2004) linking the ‘fast–slow’ plant trait gradient to the microbial community. Recently, Hennecke et al. (2023) presented a theoretical framework of how root trait gradients link with the composition of fungal communities in the rhizosphere of individual plant species. They hypothesised that root traits of a species affect the diversity, relative abundance and community structure of fungal guilds. While they found changes in the community structure of saprotrophic and plant pathogenic rhizosphere fungi, the diversity and relative abundance was not strongly correlated with root traits. However, Hennecke et al. (2023) studied plant individuals from monocultures that likely differ in the assembly mechanisms of soil fungi compared to plant communities with higher functional diversity and vegetation density (Francioli et al. 2021). Based on their framework for species‐specific effects on rhizosphere fungi (Hennecke et al. 2023), we developed hypotheses on how soil fungal guilds in bulk soil relate to community root traits in grasslands. Plant communities with dominating traits on the ‘outsourcing’ end of the collaboration gradient should have a higher diversity and relative abundance of AMF and, due to their protective role (Delavaux, Smith‐Ramesh, and Kuebbing 2017), less plant pathogenic fungal diversity and relative abundance (Semchenko et al. 2018; Wang et al. 2022). Further, plant pathogenic fungi should benefit more from higher nutrient availability and lower defence of plant tissue, both of which align with a ‘fast’ strategy of the growth‐defence trade‐off (Coley, Bryant, and Chapin 1985) of the conservation axis of root trait variation. Saprotrophic fungi strongly depend on the quality and quantity of available above‐ and belowground plant litter (Otsing et al. 2018; Wardle et al. 2004). Roots at the slow end of the conservation gradient produce low‐quality litter, yet it is unclear whether this affects saprotrophic fungal diversity and abundance (Hennecke et al. 2023).

In addition to plant functional traits, higher plant species richness influences soil fungal composition (Chen et al. 2017b; Francioli et al. 2021). Higher richness of primary producers can alter the composition and diversity of soil microbes via increased heterogeneity of resources, including roots, exudates and litter (Eisenhauer et al. 2017; Hooper et al. 2000; Steinauer, Chatzinotas, and Eisenhauer 2016). Additionally, at similar soil fertility, plant species richness is often correlated with plant cover (Hector et al. 1999) and productivity (Tilman et al. 2001), thereby increasing the amount of plant‐based resources for fungi and hence fungal biomass (Eisenhauer et al. 2017; Zak et al. 2003). Multiple studies found fungal diversity to increase with plant species richness (Dassen et al. 2017; van der Heijden et al. 1998; Yang et al. 2017), but opposing or no effects were also reported (Antoninka, Reich, and Johnson 2011; Chen et al. 2020), indicating that the plant species richness–fungal diversity relationship can depend on environmental conditions (Chen et al. 2020), scale (Eisenhauer et al. 2010) or differ between fungal guilds. Plant pathogenic fungi are predicted to be less abundant due to decreased host density with increased plant species richness (Keesing, Holt, and Ostfeld 2006). We, therefore, expect that fungal guild composition differs across the plant species richness gradient, with a stronger increase in the abundance and diversity of saprotrophic and AMF compared to plant pathogens.

In a grassland biodiversity experiment, we aimed to disentangle the effects of root traits and plant species richness on saprotrophic, plant pathogenic and AMF as the most relevant fungal guilds in grassland soils. We test three overarching hypotheses: (1) root trait organisation at the plant community‐level mirrors the RES (i.e., the collaboration and conservation gradients) previously found at the species level. (2) The diversity and relative abundance of soil fungal guilds are structured by the community RES. (3) Plant species richness is linked to increased plant biomass and thereby increases fungal diversity and biomass, but not all fungal guilds benefit equally: we expect fungal mutualists and saprotrophs to benefit more from plant species richness than plant pathogens.

Overall, we aim to advance the potential of trait‐based frameworks by integrating root functional strategies. As a first major step, we show that the root trait axes at the community level are strong determinants of the diversity and relative abundance of soil fungal guilds. Pathogenic fungi are most diverse and abundant in plant communities with ‘DIY’ and ‘fast’ root traits. Saprotrophic fungi are linked to plant species richness and both trait axes, while the diversity of AMF is only increased in plant communities with ‘outsourcing’ root traits. While both, root traits and plant species richness, are correlated with root biomass, soil fungal biomass is only driven by species richness and not root traits. Our study illustrates that root traits and plant species richness affect different properties of soil fungal communities and therefore jointly mediate the effects of plants on fungal communities.

## 2 Methods

### 2.1 Sampling Design and Soil Collection

We conducted our study at the Jena Experiment, a large‐scale long‐term biodiversity experiment. The area is located on a former arable field near the river Saale 51° N, 11° E, 135 m a.s.l. (Roscher et al. 2004). In 2002, experimental plots varying in sown plant species richness from 1 to 2, 4, 8, 16, and 60 species were set up. The plots are grouped in four blocks to account for the differences in initial soil conditions. All plots are mown twice a year and weeded manually two to three times per year. Sown and realised plant species richness are strongly positively correlated (Weisser et al. 2017).

For root trait measurements and DNA extraction, we took four soil cores (3.5 cm diameter, 5 cm depth) between May 31 and June 11 2021 in four locations across each plot. The soil cores were stored at 4°C until final preparation (no longer than 24 h after sampling). The four soil samples were pooled, and a subsample was collected for the DNA extraction and frozen at −20°C immediately. For respiration measurement and fatty acid analysis, we took four soil cores (2 cm diameter, 10 cm depth) in each plot between June 14 and 24 2021 and stored them at 4°C.

### 2.2 Root Trait Measurements

Of the 80 plots in the Jena Experiment, seven plots did not contain any of the sown plant species or did not yield enough root material to measure root traits and were therefore not further considered in our sampling. The pooled soil cores for trait measurements were soaked in water for around 15 min and then washed with tap water over a sieve and manually cleaned. Coarse roots with a diameter larger than 2 mm were manually removed from the sample. A random subset of fine roots was scanned and measured using an Epson Expression 11000XL (Epson, Tokyo, Japan) flatbed scanner at 600 dpi and the software RhizoVision Explorer (Seethepalli et al. 2021) to quantify root length, root diameter and root volume. The scanned roots were weighed, dried (48 h at 70°C) and then weighed again for dry mass. SRL was calculated as root length: dry mass and RTD as root dry mass: root volume. Fine root biomass in g/m^2^ was calculated based on the root dry mass per area of the soil cores. The roots were freeze‐dried and ground using a zirconium kit in a ball mill (MM400, Retsch, Haan, Germany). We measured root nitrogen content using two methods: for 47 random samples, relative nitrogen content (RN, % of dry weight) was quantified using an elemental analyser (Elementar vario ELII, Hanau, Germany); for the remaining 26 samples, we used a near‐infrared (NIR) spectral approach that is described in Methods S1.

The sampling of root traits from mixed‐species samples (i.e., at the community level), as also used in previous studies (Erktan et al. 2018; Prieto et al. 2015; Yang and Russo 2024), differs from the more common species‐level sampling. Because the root sample of one plant community is a mixed‐species sample on which the traits are measured directly (without species separation), only one trait value per plot/plant community is obtained. In contrast to CWM traits, measured community traits are not averaged or weighted but the community trait is determined by the natural composition of roots of different species in the sample. This method enables fast measurement of community traits while still including plasticity in species traits that would not be captured if a species were only sampled in some communities.

### 2.3 Fungal Amplicon Sequencing

We extracted genomic DNA from 0.25 to 0.3 g of thawed and homogenised soil using the Quick‐DNA Fecal/Soil Microbe Miniprep Kit (Zymo Research Europe, Freiburg, Germany) following the manufacturer's instructions. We measured the DNA content using a NanoDrop 2000c spectrophotometer (Thermo Fisher Scientific, Dreieich, Germany) and stored the DNA at −20°C until amplification. We amplified the internal transcribed spacer (ITS) region 2 using ITS4 and ITS7:ITS7o primers (Ihrmark et al. 2012; Kohout et al. 2014). Library preparation and sequencing followed the protocol described in an earlier study on rhizosphere fungi in plant monocultures in Hennecke et al. (2023). To overcome the amplification bias against AMF with ITS primers (Tedersoo et al. 2015), we used the sequence data from Albracht et al. (2024). The processing of sequences was executed using the snakemake implementation *dadasnake* (Weißbecker, Schnabel, and Heintz‐Buschart 2020) of the *DADA2* pipeline (Callahan et al. 2016). For ITS data, ASVs were then assigned to putative fungal guilds based on their taxonomic annotation and the FungalTraits database (Põlme et al. 2021). For the analysis of fungal diversity, the dataset was rarefied using the *rarefy_even_depth* function to address any potential impact of sequencing depth on ASV richness. More details on the sequencing, processing and rarefaction curves are presented in Figure S1 and Methods S2.

### 2.4 Lipid Fatty Acid and Respiration Measurement

Soil fungal biomass was determined using PLFA analysis. We extracted PLFAs from 5 g of soil following Frostegård, Tunlid, and Bååth (1991) and fractioned them into PLFAs, NLFAs and glycolipids. PLFAs and NLFAs were then measured using a gas‐chromatograph (GC‐FID Clarus 500; PerkinElmer Corporation, Norwalk, USA) with an Elite‐5 column (PerkinElmer Corporation, Norwalk, USA). PLFA and NLFA concentrations were calculated from the internal standard C19:0 (methyl nonadecanoate). Based on the classification of Ruess and Chamberlain (2010), we used 18:2ω6,9 PLFA marker as a measure of fungal biomass and the 16:1ω5 NLFA marker as a measure of AMF biomass. For the fungal: bacteria (F/B) ratio, the sum of both fungal markers was divided by the sum of the bacterial PLFA markers a15:0, i15:0, i16:0, i17:0, 16:1ω7, cy17:0 and cy19:0. For three samples, chromatograms showed very high peaks suggesting erratic measurements but could not be repeated due to small sample amount and were therefore excluded from the analysis. As recent studies raised the issue that plant biomass can also contribute to 18:2ω6,9 PLFA (Joergensen 2022), we further quantified the soil microbial biomass carbon (C_mic_) using an O_2_‐micro‐compensation apparatus (Scheu 1992). The soil was sieved at 2 mm to remove stones, large organic materials and larger organisms and added watery glucose solution to determine the maximal initial respiratory response (MIRR). C_mic_ was calculated from MIRR following Beck et al. (1997). The soil water content was estimated via drying after the end of measurements.

### 2.5 Statistical Analysis

**FIGURE 1:**
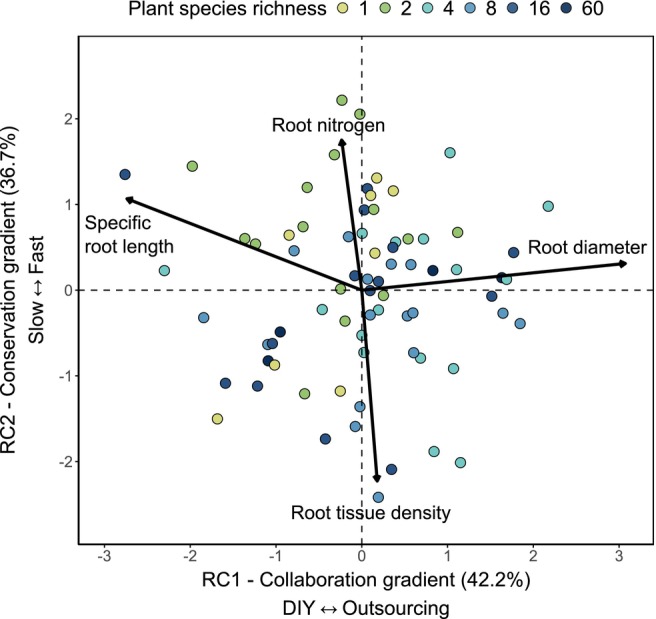
PCA of the community root traits. Each point represents a plant community (*n* = 73). The two axes closely resemble a version of the interspecific root economics space of Bergmann et al. (2020). The traits of the collaboration gradient load on the first axis, while the traits of the conservation gradient load on the second axis. Varimax rotation was used to increase interpretability of the two axes. Points are colour‐coded for plant species richness of the plot. RC, rotated component.

Data analyses were conducted in R v.4.3.2 (R Core Team 2023). We used a principal component analysis (PCA) of the community‐level root traits RD, SRL, RN and RTD, followed by varimax rotation for better interpretability of the components and inversing the scores and loadings to match the direction of trait gradients in Bergmann et al. (2020) (see Figure S2 for unrotated PCA). Rotated components (RC) 1 and 2 of the PCA captured the root economics space's main axes (Figure 1), representing the collaboration and conservation axes. Subsequent analyses used the site scores from these axes to examine the effects on fungal communities.

Fungal abundance and taxonomy were organised in a phyloseq object (McMurdie and Holmes 2013). Read numbers of ASVs with a primary lifestyle as litter saprotrophs, soil saprotrophs, wood saprotrophs and unspecified saprotrophs were summed for the total number of reads of saprotrophs. Shannon diversity was calculated within the three fungal guilds (saprotrophs, plant pathogens and AMF). Relative guild abundance was calculated as the number of reads per guild relative to the total number of reads (see Methods S2 for a broad description of the sequenced fungal community).

To test how plant species richness affected the root trait axes, we used two separate linear mixed‐effect models with RC1 and RC2 as the response and plant species richness (log) as a fixed effect and the experimental block as a random term ($\mathrm{RC}\sim \log\left(\text{plant species richness}\right)+\left(1|\text{block}\right)$). For all linear mixed models in the study, the lme4 package (Bates et al. 2015) was used and significance was assessed using type III ANOVA tables via Satterthwaite's denominator degrees of freedom method as implemented in the package lmerTest (Kuznetsova, Brockhoff, and Christensen 2017) (see Table S2 for alternative model with sequential fitting of variables). We tested how plant species richness and root trait axes are linked with the fungal guild diversity and relative abundance of each guild, as well as root biomass, PLFA and NLFA biomarker concentration, F/B ratio and soil microbial biomass carbon, using separate linear mixed‐effect models. Log‐scaled sown plant species richness and RC1 and RC2 were included as the fixed effects and the experimental block as a random term to account for any spatial effects of the field site ($\text{response}\sim \log\left(\text{plant species richness}\right)+\mathrm{RC}1+\mathrm{RC}2+\left(1|\text{block}\right)$). Effect sizes and confidence intervals were extracted using the parameter package (Lüdecke et al. 2020), using raw model variables for unstandardised effects and *z*‐transformed model variables for standardised effects. For pathogen relative abundance and root biomass, random terms did not explain any variance and linear regression was used instead.

## 3 Results and Discussion

### 3.1 Root Traits at the Community Level

To determine root traits at the community level, we sampled roots from bulk soil without separation by plant species. The PCA of these root traits shows two clear axes that explain a cumulative 79.4% of the variation (Figure 1). The trait organisation closely resembles the root economics space (RES) found at the species level across a large number of species and biomes in Bergmann et al. (2020). The first axis of the varimax‐rotated PCA, explaining 42.2% of the variation in community root traits, represents the collaboration gradient of the RES ranging from high root diameter (‘outsourcing’ strategies) to high SRL (‘do‐it‐yourself’, ‘DIY’). The second axis explained 37.2% and represents the conservation axis ranging from high root tissue density (RTD) (‘slow’) to high root nitrogen (‘fast’). This is in line with Da et al. (2023) who found the community RES to be nearly identical to the species‐level RES at global scale when using community‐weighted‐mean (CWM) root traits of woody species in a temperate forest. In contrast, Lachaise et al. (2022) identified the RES on CWM traits in observational grasslands with root nitrogen not following expected patterns. Other studies with different sets of traits measured at the community level have shown a trait coordination that partly aligns with the concept of the two RES gradients (Erktan et al. 2018; Prieto et al. 2015; Yang and Russo 2024).

Taken together, we show that directly measured plant community root traits, rather than community traits calculated from species‐specific traits, follow the same functional trade‐offs as at the species level. This finding suggests that even under the same abiotic conditions, different economic strategies of a community can be successful. It not only further demonstrates the robustness of the trait organisation across a wide plant species richness gradient but also highlights the need to better understand the conditions that lead to deviations from the RES found in other studies.

**FIGURE 2:**
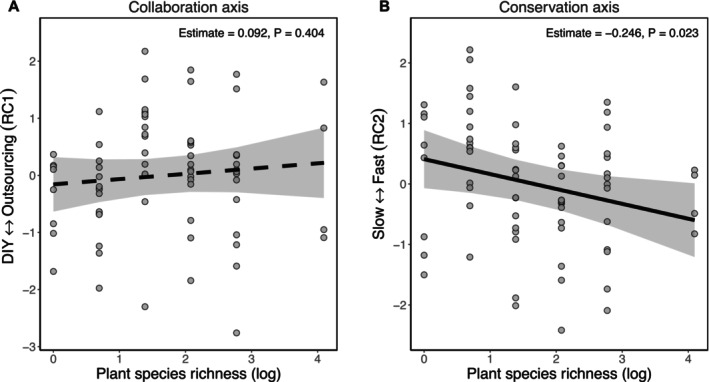
Change of root functional strategies along the plant species richness gradient. Scores of the first and second rotated components (RCs) of the root trait PCA, representing the (A) collaboration axis and (B) conservation axis, in relation to the plant species richness gradient. Each point represents the plant community of one experimental plot (*n* = 73). Regression lines are based on mixed‐effects model predictions; solid lines indicate significant relationships (*p* < 0.05); dashed lines indicate non‐significant relationships (*p* > 0.05). The grey bands around the regression lines depict the 95% confidence interval.

The community root traits varied along the gradient of sown plant species richness. While the traits of the collaboration axis, represented by the scores of the first rotated component (RC1) of the PCA, were not significantly related to plant species richness (estimate = 0.092, *p* = 0.404), scores along the conservation axis (RC2) showed a stronger relationship with plant species richness (estimate = −0.246, *p* = 0.023; Figure 2, Table S1). Accordingly, more diverse plant communities were characterised by a ‘slower’, more resource‐conservative strategy. This was driven by the decrease of root nitrogen in more diverse plant communities, which is likely an effect of intraspecific variability of root nitrogen and increased nitrogen use efficiency at high plant species richness (Mulder et al. 2002; van Ruijven and Berendse 2005).

Our plant communities are described by a single measured value per trait, rather than calculated from species‐level traits. To compare how our community‐level trait coordination reflects coordination based on species‐level traits, CWM traits would be necessary. CWM traits differ from community traits as measured in our study as traits of each species are then weighted for their proportion of the community, typically by a metric of aboveground community composition, which can differ significantly from belowground abundance (Ottaviani et al. 2020). Disproportional above‐ or belowground allocation at the species level can therefore cause a misrepresentation of CWM traits, whereas they do not affect our directly measured community traits. Further, species mean traits do not capture trait plasticity, as a species is only described by a single trait value. Given the change in root nitrogen along the plant species richness gradient, using species mean traits would distort the actual traits of the communities. Future studies using measured community traits will be critical to advance our understanding of how they correspond to species‐specific and CWM traits.

### 3.2 Links Between Root Traits and Soil Fungal Guilds

**FIGURE 3:**
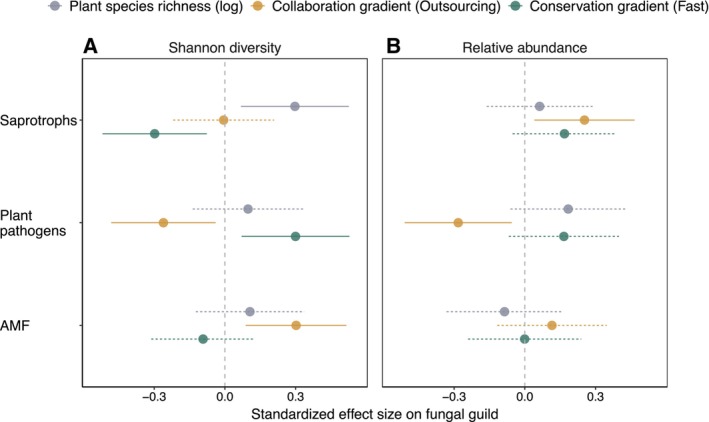
Effects of plant species richness and root trait gradients on fungal guilds. Standardised effect sizes of plant species richness (log) and root trait strategies (‘outsourcing’ along the collaboration gradient and ‘fast’ along the conservation gradient) on the Shannon diversity (A) and relative abundance (B) of saprotrophic, plant pathogenic and arbuscular mycorrhizal fungi (AMF) in bulk soil (*n* = 73). Each point represents the predicted marginal effect, with the horizontal line showing 95% confidence interval from a linear mixed‐effect model. Standardised effect sizes were extracted using z‐transformed model variables from linear mixed‐effect models.

We analysed how the diversity and relative abundance of saprotrophic, plant pathogenic and AMF were related to the sown species richness and root traits of the plant communities. By using type III sum of squares, we evaluated the effect of each factor after accounting for other model parameters; i.e., effects of root trait gradients are therefore independent of the effect of plant species richness. We found that, in line with our expectation, the Shannon diversity of fungal saprotrophs was positively related to plant species richness (Table 1, Figure 3), potentially mediated by higher root biomass (Eisenhauer et al. 2017; Mommer et al. 2015) and higher morphological and chemical diversity (Steinauer, Chatzinotas, and Eisenhauer 2016). Further, the lower litter quality of fine roots in more diverse plant communities, as indicated by the lower root nitrogen content (Chen et al. 2017a; Silver and Miya 2001), could benefit fungal saprotrophic diversity by favouring saprotrophic fungal over saprotrophic bacterial decomposers (Wardle et al. 2004). In addition to plant species richness, we hypothesised fungal saprotrophs to be dependent on the root litter quality characterised by changes in the relative nitrogen content along the conservation axis. Easily available carbon from high‐quality litter in roots with ‘fast’ traits and exudates is also used by bacteria (Hunt et al. 1987; Wardle et al. 2004) and therefore putatively less available to fungal saprotrophs, resulting in lower fungal saprotroph diversity (Table 1, Figure 3). However, we found no significant change in saprotroph relative abundance with ‘fast’ root traits, suggesting that higher‐resource quality favours fewer fungal taxa that still form similar proportions of the fungal community. ‘Outsourcing’ root strategies did not affect fungal saprotrophic diversity but significantly increased the relative abundance of saprotrophic fungi (Table 1, Figure 3). As the mechanisms behind this are not obvious and previously reported relationships between the collaboration gradient and the saprotrophic fungal community and decomposition rates are variable (Hennecke et al. 2023), we see this as an exciting avenue for further studies.

**TABLE 1:** Summary of linear mixed‐effect models testing how plant species richness and the community root trait gradients affect the Shannon diversity and relative abundance of saprotrophic, plant pathogenic and arbuscular mycorrhizal fungi (see Figure 3).

Response	Guild	Predictor	Standardised estimate	SE	*p*
Shannon diversity	Saprotrophs	Plant species richness (log)	0.297	0.115	**0.012**
		Collaboration gradient (‘outsourcing’)	−0.005	0.107	0.960
		Conservation gradient (‘fast’)	−0.298	0.111	**0.009**
	Plant pathogens	Plant species richness (log)	0.098	0.118	0.407
		Collaboration gradient (‘outsourcing’)	−0.261	0.111	**0.021**
		Conservation gradient (‘fast’)	0.299	0.115	**0.011**
	AMF	Plant species richness (log)	0.106	0.115	0.359
		Collaboration gradient (‘outsourcing’)	0.302	0.107	**0.006**
		Conservation gradient (‘fast’)	−0.093	0.110	0.403
Relative abundance	Saprotrophs	Plant species richness (log)	0.063	0.113	0.579
		Collaboration gradient (‘outsourcing’)	0.253	0.106	**0.020**
		Conservation gradient (‘fast’)	0.168	0.110	0.132
	Plant pathogens	Plant species richness (log)	0.184	0.123	0.138
		Collaboration gradient (‘outsourcing’)	−0.283	0.114	**0.015**
		Conservation gradient (‘fast’)	0.166	0.117	0.162
	AMF	Plant species richness (log)	−0.086	0.123	0.487
		Collaboration gradient (‘outsourcing’)	0.115	0.116	0.326
		Conservation gradient (‘fast’)	−0.001	0.120	0.995

*Note:* The scores of each plant community (*n* = 73) along the first and second rotated components in the root trait PCA were extracted and used as fixed effect in the model. Separate linear mixed‐effect models were used for each response variable (diversity and relative abundance of each individual guild). Each model included plant species richness and the rotated scores of the trait PCA. Standardised estimates were obtained by z‐transformation of variables prior to fitting the model. Experimental block was included as a random effect to account for spatial effects in the field site. *p*‐value < 0.05 was considered significant in bold.

Abbreviations: AMF, arbuscular mycorrhizal fungi; SE, standard error.

Plant pathogenic fungi did not change in their Shannon diversity along the plant species richness gradient (Table 1, Figure 3). A previous study in the same field site found pathogen diversity to decrease with plant species richness in old plant communities (Maciá‐Vicente et al. 2023). This is in contrast to studies on aboveground pathogens that found plant species richness to also increase pathogen richness (Rottstock et al. 2014) and indicates that pathogen dynamics belowground do not follow the same trends as aboveground. Instead, the lower‐resource quality and lower host density for specialist pathogens in diverse plant communities (Ampt et al. 2022; Wang et al. 2023) might counteract the positive effects of increased morphological and chemical diversity of roots at higher species richness, ultimately resulting in no net effect on pathogen diversity. The root trait gradients, on the other hand, were strong predictors of pathogen diversity, with ‘outsourcing’ traits along the collaboration axis and ‘slow’ traits along the conservation axis being linked with lower pathogen diversity (Table 1, Figure 3). The relative abundance of fungal pathogens also decreased with ‘outsourcing’ traits. These results are in line with our predictions and with studies that found traits of the collaboration axis at the species level to be closely related to the fungal pathogen community (Semchenko et al. 2018; Sweeney et al. 2021; Wang et al. 2022). Our results show that these effects scale from the plant species to the community level, as well as from the root or rhizosphere to bulk soil. Similar to the species level, we expect the suppression of plant pathogens by mycorrhizal symbionts to be the most likely explanation for the change along the collaboration axis (Hennecke et al. 2023). Additionally, higher root diameter itself might also be a beneficial strategy against plant pathogens, as it decreases the relative root surface (McCormack and Iversen 2019) and therefore the potential contact points with pathogens. The decreased plant investments into defence in more resource‐acquisitive plant communities along the conservation gradient (Coley, Bryant, and Chapin 1985) likely allow more plant pathogenic fungi to colonise the plant and thus be more diverse and abundant in the soil as well.

We predicted AMF to be most diverse and abundant in species‐rich plant communities and communities with ‘outsourcing’ root traits. Indeed, we found that AMF diversity was significantly linked with the collaboration axis, with higher AMF diversity found in plant communities with ‘outsourcing’ root strategies (Table 1, Figure 3). Based on the reliance of AMF on high cortex volume and root diameter (Brundrett 2002), higher intra‐radical mycorrhizal colonisation rate and higher extra‐radical hyphal length (Gryndler et al. 2006) and therefore potentially also higher abundance and diversity are expected with ‘outsourcing’ roots. Generally, AMF communities in bulk soil are more diverse than in the root, as the plant only recruits a fraction of species from the available species pool in the soil (Hempel, Renker, and Buscot 2007; Johnson et al. 2004). While we did not measure mycorrhizal colonisation in this study, the positive correlation with root diameter has been previously shown for a subset of species in our field site (Bassi et al. 2024) and our data now highlight that these trait–fungal relationships at the plant species level also transfer to AMF diversity in the soil at the plant community level. Plant species richness and the conservation axis were not related to AMF diversity (Table 1, Figure 3). The general direction of effects on the relative abundance of AMF was similar to effects on AMF diversity, but there was no significant relationship with the collaboration axis. While we calculated AMF diversity from sequence data of the AMF‐specific primers, relative abundance was calculated from the ITS2 sequence data, in which AMF only accounts for a very small portion due to the primer bias (Tedersoo et al. 2015). Whether the insignificant relationships of AMF relative abundance are due to the sequencing method or describe an actual ecological pattern needs further validation.

Overall, we found strong effects of the plant community root trait gradients on the diversity and relative abundance of fungal guilds, with each being significantly correlated with at least one trait axis. Plant species richness, however, was considerably less important than the trait axes. Specifically, we found no change in the relative abundance of any of the three fungal guilds in response to the plant species richness gradient (Table 1, Figure 3), suggesting that the fungal guild composition of the fungal community is less sensitive to the diversity of the root system and quantity and quality of plant litter input determined by plant species richness compared to root trait axes. Compared to an earlier study on rhizosphere fungi in monocultures (Hennecke et al. 2023), we found considerably stronger relationships between traits and fungal guild diversity and relative abundance in the bulk soil of plant communities. This is surprising to us, as we would have expected stronger trait effects on microbes in rhizosphere soil than in bulk soil (Lv et al. 2023). However, this might be explained by the higher plant cover and biomass in mixed plant communities (compared to the study of rhizosphere fungi in monocultures (Weisser et al. 2017)), which could amplify the effect of the plant community on soil fungi.

### 3.3 Drivers of Fungal and Microbial Biomass

Sequencing studies have substantially advanced our knowledge of the community composition of soil microbial communities and are an indispensable part of soil ecology. Yet, the increased use of compositional sequence data has partly shifted focus away from more quantitative measures of soil microbial communities. To gain a more holistic view of the effects of plant species richness and root traits on soil fungal communities, we also quantified lipid biomarkers from soil samples.

**FIGURE 4:**
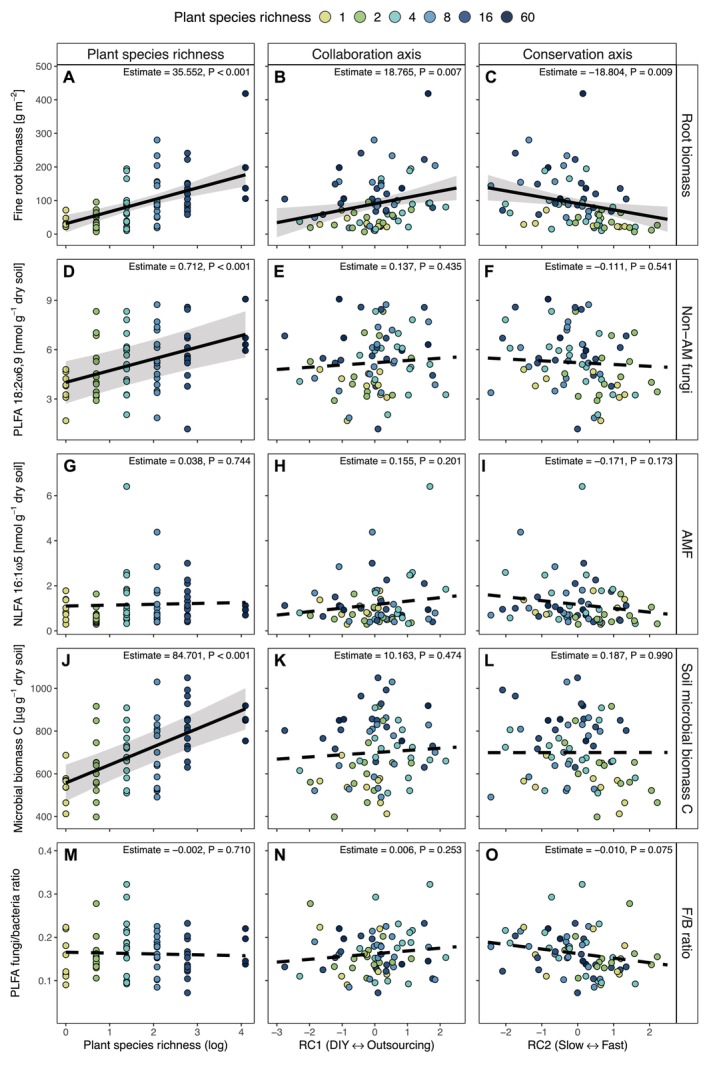
The relationship of plant species richness and root trait gradients with root biomass and soil microbial properties. Changes in root biomass (A–C), non‐arbuscular mycorrhizal (D–F) and arbuscular mycorrhizal fungi (G–I) biomarker concentration, soil microbial biomass carbon (J–L) and fungal‐to‐bacterial ratio (M–O) along the plant species richness gradient and the collaboration and conservation axis of the root economics space. Each point represents one experimental plot (*n* = 70). Regression lines are based on linear mixed‐effect model predictions; solid lines indicate significant relationships (*p* < 0.05); dashed lines indicate non‐significant relationships (*p* > 0.05). The grey bands around significant regression lines depict the 95% confidence interval.

The biomass of fine roots, a critical carbon source for the majority of soil fungi (Eisenhauer et al. 2017), increased with plant species richness but was also significantly higher in plant communities with ‘outsourcing’ and ‘slow’ root trait strategies (Table 2, Figure 4). Traits associated with these strategies (i.e., high root diameter and high RTD) generally show a positive relationship with root life span (Luke McCormack et al. 2012; Wahl and Ryser 2000) and can therefore enhance root biomass stocks. As expected, the fungal phospholipid fatty acid (PLFA) marker 18:2ω6,9, indicative of Ascomycota and Basidiomycota fungal biomass, increased strongly with plant species richness but was not associated with the collaboration and conservation axis of root traits. The soil microbial biomass carbon showed a similar positive effect of plant species richness but no effect of the root trait gradients (Table 2, Figure 4). Effects of plant species richness are potentially mediated through increased plant biomass and aboveground cover, as well as increased heterogeneity of plant‐based resources for the microbial community (Eisenhauer et al. 2017; Huang et al. 2023; Marquard et al. 2009). Contrary to our expectation, the AMF‐specific neutral lipid fatty acid (NLFA) marker 16:1ω5 was not significantly related to either plant species richness or trait axes. Given that plant species richness was previously shown to increase carbon transport to AMF (Mellado‐Vázquez et al. 2016) and that the root length colonised by AMF is correlated with AMF biomass in the soil (Barceló et al. 2020), it is surprising that the AMF biomarker did not show a positive relationship with ‘outsourcing’ root strategies and increasing plant species richness. NLFAs, unlike PLFAs, are mainly storage lipids or found in spores (Gorka et al. 2023) and are therefore not as directly relatable to fungal biomass, potentially explaining the weak effect.

**TABLE 2:** Summary of results of linear mixed‐effect models testing how plant species richness and the community root trait gradients affect root biomass, PLFA and NLFA biomarkers, soil microbial biomass carbon and the fungal:bacterial ratio (see Figure 4).

Response	Predictor	Estimate	SE	Standardised estimate	SE (in standardised units)	*p*
Fine root biomass	Plant species richness (log)	35.552	6.594	0.488	0.090	**< 0.001**
	Collaboration gradient (‘outsourcing’)	18.765	6.794	0.257	0.093	**0.007**
	Conservation gradient (‘fast’)	−18.804	7.011	−0.258	0.096	**0.009**
Non‐AM fungi PLFA (18:2ω6,9)	Plant species richness (log)	0.712	0.164	0.378	0.087	**< 0.001**
	Collaboration gradient (‘outsourcing’)	0.137	0.174	0.073	0.093	0.435
	Conservation gradient (‘fast’)	−0.111	0.181	−0.059	0.096	0.541
AMF NLFA (16:1ω5)	Plant species richness (log)	0.038	0.116	0.039	0.119	0.744
	Collaboration gradient (‘outsourcing’)	0.155	0.120	0.159	0.123	0.201
	Conservation gradient (‘fast’)	−0.171	0.124	−0.176	0.127	0.173
Soil microbial biomass carbon	Plant species richness (log)	84.701	13.316	0.543	0.085	**< 0.001**
	Collaboration gradient (‘outsourcing’)	10.163	14.115	0.065	0.091	0.474
	Conservation gradient (‘fast’)	0.187	14.618	0.001	0.094	0.990
Fungal: bacterial ratio	Plant species richness (log)	−0.002	0.005	−0.040	0.108	0.710
	Collaboration gradient (‘outsourcing’)	0.006	0.006	0.131	0.114	0.253
	Conservation gradient (‘fast’)	−0.010	0.006	−0.213	0.118	0.075

*Note:* The scores of each plant community (*n* = 70) along the first and second rotated components in the root trait PCA were extracted and used as fixed effect in the model. Separate linear mixed‐effect models were used for each response variable. Each model included plant species richness and the rotated scores of the trait PCA. Standardised estimates were obtained by z‐transformation of variables prior to fitting the model. Experimental block was included as a random effect to account for spatial effects in the field site. *p*‐value < 0.05 was considered significant in bold.

Abbreviations: AMF, arbuscular mycorrhizal fungi; NLFA, neutral lipid fatty acid; non‐AM, non‐arbuscular mycorrhizal; PLFAs, phospholipid fatty acids; SE, standard error.

The ratio between fungal and bacterial biomass (F/B) is considered a proxy for nutrient cycling rates in soils as a higher fungal proportion decreases nutrient cycling rates and increases nutrient retention compared to bacterial‐dominated communities (Bardgett 2017; Wardle et al. 2004). We found no change in the F/B ratio along the plant species richness gradient and the collaboration axis but a marginally significant decrease with ‘fast’ root traits along the conservation axis (Table 2, Figure 4). This aligns with previous concepts and results suggesting that bacteria benefit from the higher litter quality of ‘fast’ above‐ and belowground traits (Bardgett 2017).

Generally, the quantification of absolute abundances or biomass of individual fungal guilds is not possible in the same way as for relative abundances. Approaches using qPCR methods can quantify gene copies under certain circumstances but are sensitive to biases during the DNA extraction or require standardisation (Baldrian et al. 2013; Feinstein, Sul, and Blackwood 2009). Since the PLFA biomarker 18:2ω6,9 is largely determined by the biomass of Ascomycota and Basidiomycota (Klamer and Bååth 2004), we used it as an indicator of fungal biomass of non‐AMF, which include saprotrophic as well as pathogenic fungi. Because PLFA biomarkers and sequencing rely on very different components of the fungal community (lipid of cell membrane compared to DNA in the nucleus), the results are not directly comparable. However, under the assumption that both methods similarly characterise the fungal community, we would estimate the absolute abundance of each guild by multiplying its relative sequence abundance with the absolute fungal biomass. In this case, the root trait axes would not affect the absolute fungal guild abundance any different from the relative guild abundance, as the absolute fungal biomass is not correlated with the traits. Due to the uncertainty of the approach, we refrain from doing this here. However, studies using a combined approach, by including relative (e.g., sequencing) and absolute measures (e.g., PLFA and respiration), provide valuable opportunities to overcome the limitations of the compositional nature of sequencing data in the absence of appropriate qPCR methods.

### 3.4 Further Considerations

Collecting data in a biodiversity experiment allows us to separate the effects of plant species richness and traits. Only basing research on observational studies can be misleading due to potential unexplored confounding factors (Eisenhauer et al. 2016, 2024). However, biodiversity experiments inevitably involve management practices such as weeding to maintain the species composition. Our experimental management is applied in the most careful way possible, and it has been shown that weeding disturbances cannot explain plant diversity effects on ecosystem functions (Weisser et al. 2017). Significant plant diversity effects on soil communities and ecosystem functions have also been found in short‐term microcosm experiments with standardised plant density across diversity levels and without any weeding (Eisenhauer et al. 2017). The research field has carefully checked if plant diversity effects that are generally found in these experiments are realistic and not due to unrealistic species combinations (Jochum et al. 2020). Our manuscript builds on the value of the long‐term existence of plant diversity and trait gradients to reveal hitherto hidden interactions with soil‐borne fungi as key components of the living soil. Careful comparisons between our experimental setup and observational studies are required to validate the relevance of our findings for natural plant communities (Oelmann et al. 2021).

### 3.5 Conclusions

Our study demonstrates that in experimental grassland communities, fine root functional strategies of the root economics space scale from the species level to the community level. We further demonstrate that these functional strategies of plant communities structure the guild composition of soil fungal communities, with saprotrophic, plant pathogenic and arbuscular mycorrhizal varying in diversity or relative abundance depending on the root traits of the plant community. In line with our expectation, ‘fast’ root traits are associated with lower saprotroph diversity and higher plant pathogen diversity. ‘Outsourcing’ root traits are linked with higher relative abundance of saprotroph and lower diversity and relative abundance of plant pathogens. Plant species richness, however, is only a weak driver of the fungal and microbial community composition but instead drives microbial and fungal biomass in the soil (Figures 3 and 4). Ultimately, root trait gradients structure the soil fungal guild composition, but plant species richness drives fungal biomass. These contrasting results on the role of plant species richness and root trait gradients highlight that a diversity of mechanisms needs to be considered in future predictions of how changes in plant communities will affect soil biodiversity and functioning.

## Author Contributions

J.H., J.B., N.E., A.H.‐B., T.W.K., M.L., L.M. and A.W. conceived the idea of the study. J.H., L.B., C.A., A.A., A.F., L.H., Y.P.A.d.S., A.R. and M.D.S. collected the data; J.H. and A.H.‐B. processed the sequence data, and J.H. analysed the data. J.H. led the writing of the manuscript, and all authors contributed to reviewing and editing.

## Conflicts of Interest

The authors declare no conflicts of interest.

### Peer Review

The peer review history for this article is available at https://www.webofscience.com/api/gateway/wos/peer‐review/10.1111/ele.70032.

## Supporting information


Data S1.

## Data Availability

The data and code associated with this study are available from https://doi.org/10.5281/zenodo.13990357. The raw Illumina sequences generated in this study are available in the NCBI Sequence Read Archive (BioProject: PRJNA1074103 and PRJNA988299).
