# Supplementary material for: Plant Species Richness and the Root Economics Space Drive Soil Fungal Communities
Source: Ecol Lett. 2024 Dec 31;28(1):e70032. doi: 10.1111/ele.70032 (PMC11687415; doi:10.1111/ele.70032)
Supplement: Supplementary file 1 — Data S1. [file ELE-28-0-s001.docx]

**Supplementary Information**

Plant species richness and the root economics space drive soil fungal communities

Justus Hennecke^1,2,*^, Leonardo Bassi^1,2^, Cynthia Albracht^3,4,5^, Angelos Amyntas^2,6,7^, Joana Bergmann^8^, Nico Eisenhauer^2,9^, Aaron Fox^10,11^ Lea Heimbold^12,2^, Anna Heintz-Buschart^3^, Thomas W. Kuyper^13^, Markus Lange^14^, Yuri Pinheiro Alves de Souza^10,15^, Akanksha Rai^14^, Marcel Dominik Solbach^16^, Liesje Mommer^17^& Alexandra Weigelt^1,2^

^1^ Systematic Botany and Functional Biodiversity, Institute of Biology, Leipzig University, 04103 Leipzig, Germany

^2^ German Centre for Integrative Biodiversity Research (iDiv) Halle-Jena-Leipzig, 04103 Leipzig, Germany

^3^ Biosystems Data Analysis, Swammerdam Institute for Life Sciences, University of Amsterdam, 1098XH Amsterdam, the Netherlands

^4^ Department Soil Ecology, Helmholtz Centre for Environmental Research – UFZ, 06120 Halle (Saale), Germany

^5^ Institute for Biosafety in Plant Biotechnology, Julius Kühn Institute, 06484 Quedlinburg, Germany

^6^ Institute of Biodiversity, Friedrich Schiller University Jena, 07743 Jena, Germany

^7^ J.F. Blumenbach Institute of Zoology and Anthropology, University of Göttingen, 37073 Göttingen, Germany

^8^ Leibniz Centre for Agricultural Landscape Research (ZALF), 15374 Müncheberg, Germany

^9^ Institute of Biology, Leipzig University, 04103 Leipzig, Germany

^10^ Chair of Environmental Microbiology, TUM School of Life Science, Technical University of Munich, 85354 Freising, Germany

^11^ Environment, Soils and Land Use, Teagasc, Johnstown Castle, Co. Wexford, Y35HK54, Ireland.

^12^ Institute of Biology, Martin-Luther-University Halle-Wittenberg, 06120 Halle (Saale), Germany

^13^ Soil Biology Group, Wageningen University, 6708 PB Wageningen, the Netherlands

^14^ Department of Biogeochemical Processes, Max Planck Institute for Biogeochemistry, 07745 Jena, Germany

^15^ Research Unit Comparative Microbiome Analysis, Helmholtz Zentrum München, 85764 Neuherberg, Germany

^16^ Terrestrial Ecology Group, Institute of Zoology, University of Cologne, 50674 Cologne, Germany

^17^ Forest Ecology and Forest Management Group, Wageningen University & Research, PO box 47, 6700 AA Wageningen, the Netherlands

**Supplementary Table S1: Summary of ANOVA results for the effect of plant species richness on the rotated components of the root trait PCA and on the individual fine root traits.** The scores of each plant community (n = 73) along the first and second rotated component (RC) in the root trait PCA were extracted and used as response in the model. Experimental block was included as a random effect to account for spatial effects in the field site. df_error_, error degrees of freedom; SE, standard error.

|  | **Effect of plant species richness (log)** | | | | |
| --- | --- | --- | --- | --- | --- |
| **Response** | **df_error_** | **Coefficient** | **SE** | ***F*** | ***p*** |
| RC1 (Collaboration gradient) | 68.301 | 0.092 | 0.110 | 0.704 | 0.404 |
| RC2 (Conservation gradient) | 68.416 | -0.246 | 0.106 | 5.412 | **0.023** |
|  |  |  |  |  |  |
| Root diameter | 72.000 | 0.004 | 0.004 | 0.925 | 0.339 |
| Specific root length | 69.180 | -11.678 | 8.121 | 2.068 | 0.155 |
| Root nitrogen | 71.000 | -0.176 | 0.046 | 14.417 | **<0.001** |
| Root tissue density | 69.328 | 0.003 | 0.003 | 0.8616 | 0.357 |

**Supplementary Table S2: Summary of ANOVA with type 1 sum-of-squares results for fungal diversity and relative abundance of guilds.** The scores of each plant community (n = 73) along the first and second rotated axis in the root trait PCA were extracted and used as fixed effect in the model. Experimental block was included as a random effect to account for spatial effects in the field site. AMF, arbuscular mycorrhizal fungi; df_error_, error degrees of freedom; SE, standard error.

| **Response** | **Guild** | **Predictor** | **df** | **df_error_** | ***F*** | ***p*** |
| --- | --- | --- | --- | --- | --- | --- |
| Shannon diversity | Saprotrophs | Plant species richness (log) | 1 | 66.615 | 11.589 | **0.001** |
|  |  | Collaboration gradient (‘outsourcing’) | 1 | 68.995 | 0.013 | 0.908 |
|  |  | Conservation gradient (‘fast’) | 1 | 68.798 | 7.231 | **0.009** |
|  | Plant pathogens | Plant species richness (log) | 1 | 66.236 | 0.003 | 0.960 |
|  |  | Collaboration gradient (‘outsourcing’) | 1 | 68.542 | 5.248 | **0.025** |
|  |  | Conservation gradient (‘fast’) | 1 | 68.767 | 6.824 | **0.011** |
|  | AMF | Plant species richness (log) | 1 | 66.533 | 2.079 | 0.154 |
|  |  | Collaboration gradient (‘outsourcing’) | 1 | 67.964 | 7.861 | **0.007** |
|  |  | Conservation gradient (‘fast’) | 1 | 66.026 | 0.708 | 0.403 |
| Relative abundance | Saprotrophs | Plant species richness (log) | 1 | 66.136 | 0.162 | 0.689 |
|  |  | Collaboration gradient (‘outsourcing’) | 1 | 67.629 | 5.832 | **0.018** |
|  |  | Conservation gradient (‘fast’) | 1 | 67.830 | 2.323 | 0.132 |
|  | Plant pathogens | Plant species richness (log) | 1 | 69.000 | 0.917 | 0.342 |
|  |  | Collaboration gradient (‘outsourcing’) | 1 | 69.000 | 5.996 | **0.017** |
|  |  | Conservation gradient (‘fast’) | 1 | 69.000 | 2.001 | 0.162 |
|  | AMF | Plant species richness (log) | 1 | 66.209 | 0.402 | 0.528 |
|  |  | Collaboration gradient (‘outsourcing’) | 1 | 68.333 | 0.981 | 0.326 |
|  |  | Conservation gradient (‘fast’) | 1 | 68.572 | 0.000 | 0.995 |


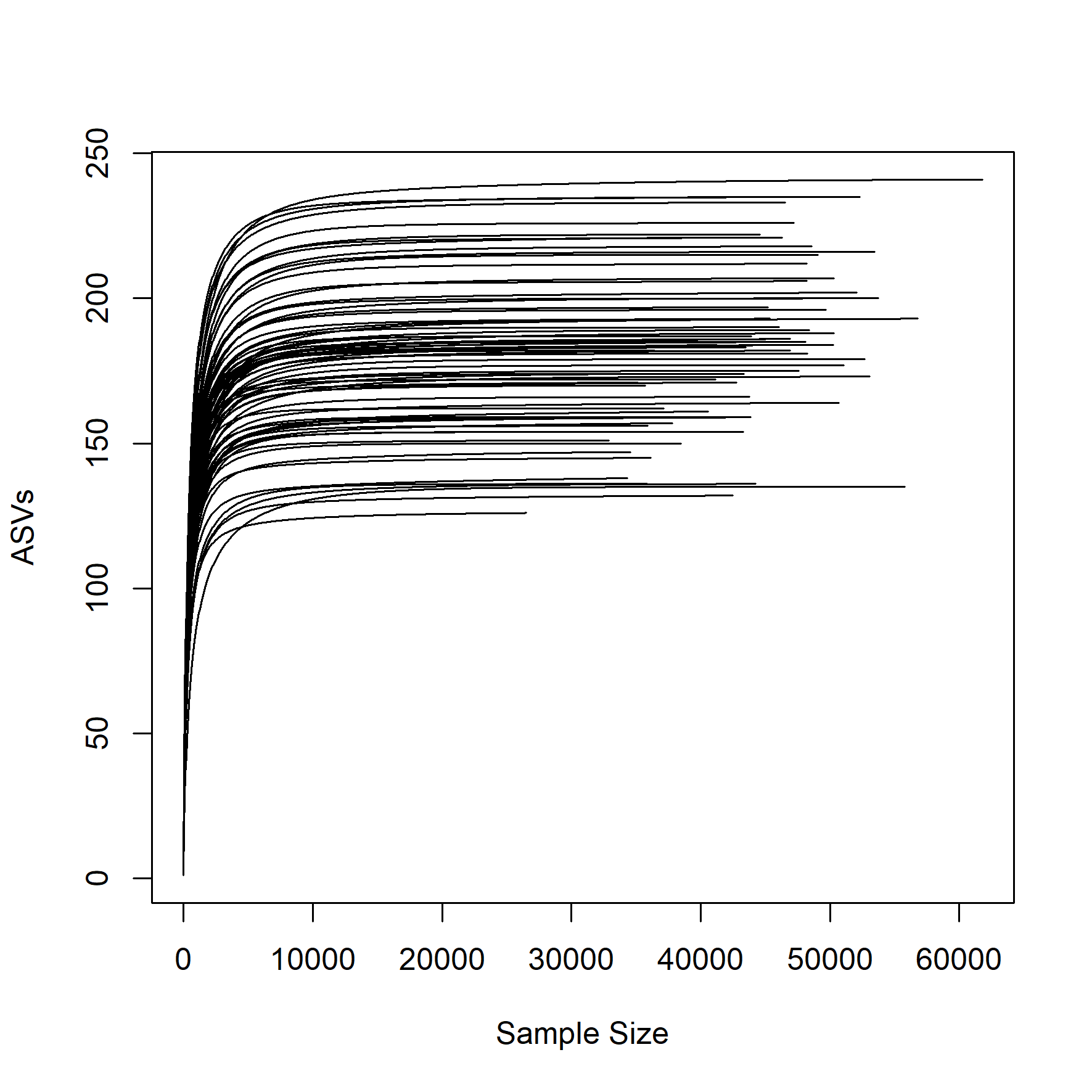


**Supplementary Fig. S1: Rarefaction curves of the ITS2 sequence data.** Each line represents one of the 73 samples, showing increasing number of amplicon sequence variants (ASVs) with the number of reads until saturation is reached.

**
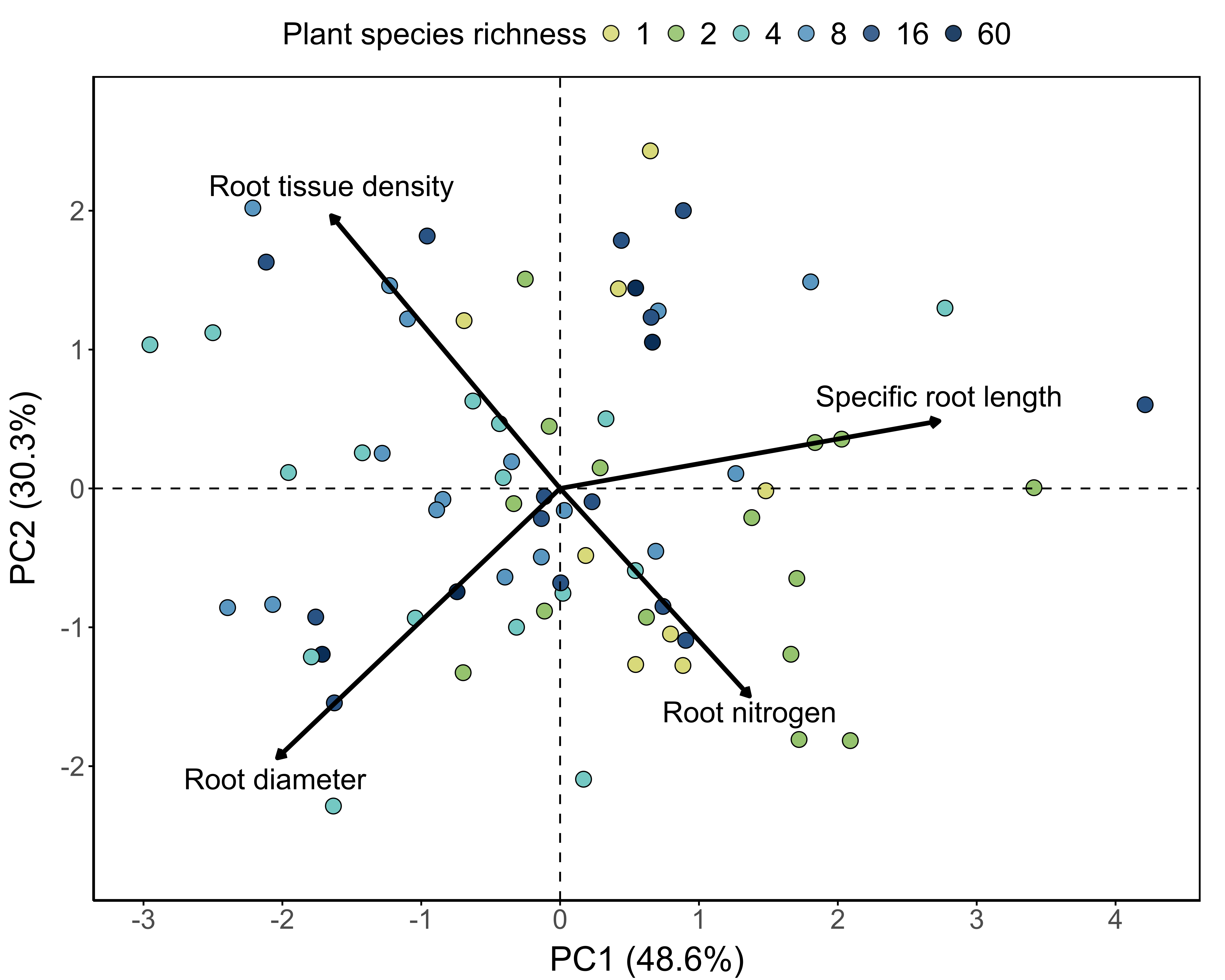
 Supplementary Fig. S2: Unrotated PCA of the community root traits.** Each point represents a plant community (*n* = 73). Points are color-coded for plant species richness of the plot. RC, rotated component.

**Supplementary Methods S1: Nitrogen measurement using near-infrared spectroscopy (NIR)**

Milled root samples were freeze-dried again to measure NIR in the range of 9090–4000cm^−1^ at 8cm^−1^ resolution in transmission mode (Multi-Purpose FT-NIR-Analyzer, Bruker Corporation, Billerica, USA). For each sample, five independent measurements were averaged. We converted transmission to absorbance as log_10_(1/Transmission) and used it in combination with a bootstrapped CARS-PLSR procedure(Richter & Bassi 2023) to predict nitrogen content. These 26 samples were distributed among plots with 1, 2, 4, 8, 16 and 60 species plots with 2, 1, 3, 9, 8 and 3 samples, respectively.

**Supplementary Methods S2: Amplicon sequencing and description of ITS2 dataset**

After PCR, three positive PCR products were pooled, purified with AMPure XP beads (Beckman Coulter), and indexed with the Nextera XT Illumina Index Kit (Illumina Inc., San Diego, USA). The samples were then pooled to equal molarity and the paired-end sequencing of 2 × 300 bp was performed using a MiSeq Reagent Kit v3 on an Illumina MiSeq System at the Department of Soil Ecology of the Helmholtz-Centre for Environmental Research (UFZ, Halle/Saale, Germany). To overcome the amplification bias against AMF with ITS primers (Tedersoo *et al.* 2015), we used the sequence data from Albracht *et al.* (2024). In short, the SSU of AMF was amplified using a nested PCR with primer pair Glomer 1536 / WT0 for the first PCR and NS31 / AML2 for the second PCR according to Wahdan *et al.* (2021) and the sequencing was performed as for the ITS2 sequencing. For a detailed description of the sequencing of AMF communities we refer to Albracht *et al.*(2024)*.*

To summarize, *cutadapt*(Martin 2011) was used to trim the primer sequences from raw reads. Quality trimming was performed with a minimum length of 70 bp for ITS and 260 bp (fwd) / 210 bp (rvs) for AMF, truncation of reads at positions with a PHRED score below 15 for ITS, and exclusion of reads with an expected error higher than 2. The identification of exact sequence variants (Amplicon Sequence Variants, ASVs) included merging read pairs with a minimum overlap of 15 bp (ITS) and 12 bp (AMF) and a maximum of three (ITS) and zero (AMF) mismatches. Chimeras were filtered using DADA2's 'consensus' algorithm. Taxonomic classification was conducted using the *mother* (Schloss *et al.* 2009) implementation of the Bayesian classifier against the UNITE v8.2 database (Nilsson *et al.* 2019) (ITS) and SILVA v138 SSUref database (Quast *et al.* 2013) for AMF. Non-fungal ASVs (for ITS sequences) and non-Glomeromycotinian ASVs (for AMF sequences) were discarded. For ITS data, ASVs were then assigned to putative fungal guilds based on their taxonomic annotation and the FungalTraits database (Põlme *et al.* 2021). Further, all Glomeromycotinian ASVs in the ITS data were assigned to be arbuscular mycorrhizal. For the AMF data, ASVs were blasted against the MaarjAM database (Öpik *et al.* 2010) and assigned to virtual taxa (VTX). ASVs without VTX assignment were extracted, singletons removed, and used for the construction of a maximum likelihood phylogenetic tree based on a general time-reversible, discrete gamma (GTR+G) model using raxML (Stamatakis 2014) and FasttreeMP (Price *et al.* 2010). Consequently, these ASVs were associated with custom virtual taxa (VTC) characterized by cophenetic distances below 0.03.

Our filtered ITS2 sequence dataset of 73 samples consisted of 3,251,726 fungal sequence reads, with a mean of 44,544 reads per sample (min. 26,494; max. 61,786 reads per sample). These were grouped into 5,276 ASVs, of which 4,472 were taxonomically assigned to the phylum level. Of these, Ascomycota (73.80%) had the most sequence reads, followed by Basidiomycota (12.07%), Mortierellomycota (11.43%) and Chytridiomycota (2.12%). A total of 2,309 ASVs (43.76%, corresponding to 64.09% of reads) could be assigned to at least one guild in the FungalTraits database (Põlme *et al.* 2021). Of these, saprotrophs (which include soil saprotrophs, litter saprotrophs, wood saprotrophs, and unspecified saprotrophs) made up 58.34%, plant pathogens 25.82%, and arbuscular mycorrhiza 0.30% of reads.

**References of Supplementary Methods:**

Albracht, C., Solbach, M.D., Hennecke, J., Bassi, L., van der Ploeg, G.R., Eisenhauer, N., *et al.* (2024). Common soil history is more important than plant history for arbuscular mycorrhizal community assembly in an experimental grassland diversity gradient. *Biol. Fertil. Soils*, 60, 547–562.

Martin, M. (2011). Cutadapt removes adapter sequences from high-throughput sequencing reads. *EMBnet.journal*, 17, 10–12.

Nilsson, R.H., Larsson, K.-H., Taylor, A.F.S., Bengtsson-Palme, J., Jeppesen, T.S., Schigel, D., *et al.* (2019). The UNITE database for molecular identification of fungi: handling dark taxa and parallel taxonomic classifications. *Nucleic Acids Res.*, 47, D259–D264.

Öpik, M., Vanatoa, A., Vanatoa, E., Moora, M., Davison, J., Kalwij, J.M., *et al.* (2010). The online database MaarjAM reveals global and ecosystemic distribution patterns in arbuscular mycorrhizal fungi (Glomeromycota). *New Phytol.*, 188, 223–241.

Põlme, S., Abarenkov, K., Henrik Nilsson, R., Lindahl, B.D., Clemmensen, K.E., Kauserud, H., *et al.* (2021). FungalTraits: a user-friendly traits database of fungi and fungus-like stramenopiles. *Fungal Divers.*, 105, 1–16.

Price, M.N., Dehal, P.S. & Arkin, A.P. (2010). FastTree 2 – Approximately Maximum-Likelihood Trees for Large Alignments. *PLOS ONE*, 5, e9490.

Quast, C., Pruesse, E., Yilmaz, P., Gerken, J., Schweer, T., Yarza, P., *et al.* (2013). The SILVA ribosomal RNA gene database project: improved data processing and web-based tools. *Nucleic Acids Res.*, 41, D590–D596.

Richter, R. & Bassi, L. (2023). Bagging-CARS-PLS model R code.

Schloss, P.D., Westcott, S.L., Ryabin, T., Hall, J.R., Hartmann, M., Hollister, E.B., *et al.* (2009). Introducing mothur: Open-source, platform-independent, community-supported software for describing and comparing microbial communities. *Appl. Environ. Microbiol.*, 75, 7537–7541.

Stamatakis, A. (2014). RAxML version 8: a tool for phylogenetic analysis and post-analysis of large phylogenies. *Bioinformatics*, 30, 1312–1313.

Tedersoo, L., Anslan, S., Bahram, M., Põlme, S., Riit, T., Liiv, I., *et al.* (2015). Shotgun metagenomes and multiple primer pair-barcode combinations of amplicons reveal biases in metabarcoding analyses of fungi. *MycoKeys*, 10, 1–43.

Wahdan, S.F.M., Reitz, T., Heintz-Buschart, A., Schädler, M., Roscher, C., Breitkreuz, C., *et al.* (2021). Organic agricultural practice enhances arbuscular mycorrhizal symbiosis in correspondence to soil warming and altered precipitation patterns. *Environ. Microbiol.*, 23, 6163–6176.
